# Retrospective Study on Etiology and Management of Epistaxis in a Tertiary Care Hospital

**DOI:** 10.7759/cureus.104718

**Published:** 2026-03-05

**Authors:** Meenu P.S, Usha G, Bosco Suriya Luke Rathnakumar, S.M. Azeem Mohiyuddin

**Affiliations:** 1 Otolaryngology - Head and Neck Surgery, Sri Devaraj Urs Academy of Higher Education & Research, Kolar, IND; 2 Otolaryngology, Sri Devaraj Urs Academy of Higher Education & Research, Kolar, IND

**Keywords:** anterior nasal packing, epistaxis, hypertension, posterior nasal packing, trauma

## Abstract

Background

Epistaxis is one of the most common emergencies encountered across all age groups. However, its etiological patterns and management outcomes exhibit significant regional variation. This retrospective study aims to assess the epidemiology, etiology, clinical presentation, treatment modalities, and outcomes of epistaxis in a tertiary referral hospital.

Methods

A retrospective, observational study was conducted at our center involving 384 patients who presented with epistaxis between January 2020 and June 2025. Patient records were reviewed for demographics, etiology, treatment modalities, and outcomes. Descriptive statistics were used to summarize the data.

Results

Anterior epistaxis constituted 68.7% (264/384) of cases. Trauma was the most common etiology (59.9%, 230/384), followed by hypertension (20.1%, 77/384) and dengue fever (5.9%, 23/384). The most common intervention was anterior nasal packing (59.9%, 230/384), followed by conservative management (26.6%, 102/384). Cauterization was used in 5.9% (23/384) of cases, and combined anterior and posterior packing in 6.3% (24/384). Surgical ligation was rarely required (1.3%, 5/384). Initial bleeding control was achieved in 93.7% (360/384) of patients, and 6.3% (24/384) required repeat intervention. Documented complications included re-bleeding (5.9%), sinusitis (3.9%), and septal ulceration (2.1%).

Conclusion

Trauma and hypertension were the primary etiologies of epistaxis, with anterior nasal packing constituting the fundamental approach to management. These results emphasize the significance of targeted prevention, early etiological identification, and structured management protocols to enhance outcomes.

## Introduction

Epistaxis is one of the most common otolaryngological emergencies, with nearly 60% of individuals experiencing at least one episode during their lifetime and about 6%-10% requiring clinical intervention [1].

Recent advances in diagnostic and therapeutic modalities have improved the efficacy of management of epistaxis. However, considerable variation persists across institutions depending on patient demographics, infrastructure, and available expertise.

Retrospective analyses of cases of epistaxis in a tertiary care center are crucial for understanding disease patterns, etiological profiles, severity distribution, and treatment outcomes. Such evaluations support evidence-based decision-making, help identify modifiable risk factors, and strengthen triage and management protocols tailored to regional populations.

As tertiary care facilities function as referral centers for complex or refractory cases, they offer an ideal setup for assessing varied etiologies and advanced therapeutic practices. A retrospective study in such a setting can yield essential data on the prevalence of epistaxis, demographic characteristics, recurrence patterns, etiological distribution, and the relative effectiveness of various interventions. Additionally, evaluating treatment outcomes - including complications, rebleeding rates, and length of hospital stay - provides insight into the quality of care and opportunities for improvement.

## Materials and methods

Aim

This retrospective study aims to contribute evidence on the etiological spectrum and management trends of epistaxis in a tertiary care setting, ultimately supporting improved clinical outcomes and optimized use of healthcare resources.

Objectives

The objectives of this study are to (1) analyze the etiological factors for epistaxis, (2) assess the frequency and distribution of different causes across age and gender groups, (3) evaluate various treatment modalities employed in the management of epistaxis, and (4) study the treatment outcomes and identify commonly used interventions in the hospital setting.

Materials and methods

A retrospective, observational study was carried out at a tertiary referral center between 1st January 2020 and 30th June 2025, with prior approval from the institutional ethics committee (Ref: SDUAHER/R&D/CEC/SDUMC-PG/265/NF/-2025-26). All cases of epistaxis documented during this period were screened for eligibility.

Inclusion criteria

All patients who presented with epistaxis and whose records contained sufficient clinical and treatment-related information during the study period were included.

Exclusion criteria

Patient’s file with incomplete documentation lacking essential data such as demographic details, etiology, key investigations, treatment administered, or outcome was excluded.

Methodology

A retrospective review of hospital records for all patients admitted with a diagnosis of epistaxis was conducted during the set period. A total of 653 patient records were initially identified. Records were screened for eligibility, and patients were excluded if they had incomplete documentation (n=144), absconded during treatment (n=22), or requested discharge against medical advice (n=103). After applying these exclusion criteria, a total of 384 patients with complete and detailed clinical documentation were included in the final analysis.

Data was analyzed using SPSS software version 22 (IBM Corp., Armonk, NY). Descriptive statistics such as frequency and percentage were used for qualitative variables, while quantitative variables were summarized using the mean and standard deviation. No inferential statistical testing was performed, as the study was descriptive in nature.

## Results

Most patients were in the 41-60 years age group. Males constituted 76.3% of cases and females 23.7%. Anterior epistaxis was more common than posterior, accounting for 68.7% of cases, as shown in Table 1. 

**Table 1:** Distribution of patients by age, gender, and type of epistaxis

Parameter	Subcategory	Number of patients	Percentage (%)
Age group (years)	0-20	52	13.5
	21-40	118	30.7
	41-60	145	37.8
	>60	69	18.0
Gender distribution	Male	293	76.3
	Female	91	23.7
Type of epistaxis	Anterior	264	68.7
	Posterior	120	31.3
Total	384		

Trauma was the leading cause (60%), followed by hypertension (20%). Less common causes included dengue, coagulopathies, drug-induced bleeding, idiopathic cases, and neoplasms, as indicated in Table 2.

**Table 2:** Etiological factors of epistaxis

Etiology	Number	Percentage (%)
Trauma	230	59.9
Hypertension	77	20.1
Dengue	23	5.9
Coagulopathies	12	3.1
Drug-induced	18	4.7
Idiopathic	18	4.7
Neoplastic	6	1.6
Total	384	

Among the 384 patients, 150 (39.1%) presented with a single episode of epistaxis, while 96 (25.0%) had recurrent episodes. Associated symptoms were documented for 234 patients. Table 3 presents symptom counts among the 234 patients, and percentages are shown using the full study cohort as the denominator (N=384) for clarity.

**Table 3:** Associated symptoms Patients who presented with epistaxis and associated symptoms, n=234 Patients who presented with epistaxis as the only symptom, n=150 Total cohort, n=384

Presentation	Number	Percentage (%)
Recurrent episodes	96	25
Nasal obstruction	47	12.2
Headache	62	16.1
Generalized fatigue	29	7.5
Total	234	

Anterior nasal packing was the most frequently used intervention (59.9%). Conservative management accounted for 26.6% of cases, while combined packing and cauterization were used in 6.3% and 5.9% of cases, respectively. Surgical ligation was rarely required (1.3%), as shown in Table 4.

**Table 4:** Treatment modalities used

Treatment modality	Number	Percentage (%)
Conservative management	102	26.6
Anterior nasal packing	230	59.9
Combined nasal packing	24	6.3
Cauterization	23	5.9
Surgical ligation	5	1.3
Total	384	

Initial bleeding control was achieved in 93.7% of patients. Only 6.3% required repeat intervention, as shown in Table 5. Blood transfusion was required in less than 1% of cases.

**Table 5:** Treatment outcomes

Outcome	Number	Percentage (%)
Successful control initially	360	93.7
Required repeat intervention	24	6.3
Total	384	

Re-bleeding was the most common complication (5.9%), suggesting that a small proportion required further management. Sinusitis and septal ulceration occurred in a minority of cases, likely related to nasal packing and mucosal irritation, as shown in Table 6.

**Table 6:** Complications observed

Complication	Number	Percentage (%)
Sinusitis	15	3.9
Septal ulceration	8	2.1
Re-bleeding	23	5.9
Total	46	

Nearly half of the patients stayed for two to three days, representing the typical observation period for epistaxis management. A short stay of one day was sufficient in 34.4% of cases, while a prolonged admission for four to six days was needed only in more severe or complex cases, as indicated in Table 7.

**Table 7:** Duration of hospital stay

Duration (days)	Number	Percentage (%)
1 day	132	34.4
2-3 days	187	48.7
4-6 days	65	16.9
Total	384	

Trauma-related epistaxis was most commonly managed with anterior nasal packing. Among patients with hypertension, combined packing was used in some cases. As this was a descriptive study without inferential testing, these observations should not be interpreted as statistically supported subgroup differences.

## Discussion

This study provides a comprehensive evaluation of the demographic profile, etiological spectrum, and treatment outcomes of epistaxis in a tertiary care setting, and the findings closely align with several previous studies conducted across different geographical regions [1-5]. In our study, the highest proportion of patients belonged to the age group of 41-60 years (37.8%), followed by 21-40 years (30.7%), reflecting a middle-aged predominance. This pattern partially correlates with the findings of Islam et al. [4], who reported that the majority of cases occurred in the second (21.15%) and sixth decades (19.23%) of life, indicating that age distribution varies across populations. While Gilyoma et al. [5] observed a modal age group of 31-40 years, suggesting that young adults also form a large proportion of cases in certain settings.

Our male-to-female ratio of 3.2:1 (76.3% males) parallels the trends reported by Islam et al. [4], who documented a ratio of 2.47:1, and Gilyoma et al. [5], who documented an even higher male predominance of 3.4:1, while Sharma et al. [6] found a ratio of 2.6:1 with a predominance of epistaxis incidence in elderly patients. Adoga et al. found the male-to-female preponderance of 2.3:1, with a predilection for patients in the third decade [7]. These consistent findings across studies confirm that epistaxis predominantly affects males, possibly due to increased exposure to trauma and occupational hazards.

In terms of the type of bleeding, our study found anterior epistaxis in 68.7% of cases and posterior bleeding in 31.3%. This aligns with similar other studies found in the literature such as Islam et al., [4] where traumatic groups predominantly demonstrated anterior epistaxis (69.57%) and posterior epistaxis was more common in non-traumatic causes (46.55%), and Gilyoma et al., where 88.7% of cases were of anterior bleeds, followed by 11.3% cases of posterior bleeds [5], supporting the observation that anterior epistaxis significantly outweighs posterior forms in most settings. At the same time, other studies reported 47.9% vs. 15.07% and 69.29 vs. 30.71%, respectively [8,9].

The etiological distribution in our study demonstrated trauma as the most common cause (60%), followed by hypertension (20%), dengue (6%), coagulopathies (3%), drug-induced causes (4.7%), idiopathic causes (4.7%), and neoplasms (1.6%).

Trauma was also the leading cause in several earlier studies; for instance, Islam et al. reported traumatic epistaxis in 44.23% of patients, while Gilyoma et al. and Akinpelu et al. [5,10] reported 70.9% of cases, particularly those resulting from road traffic accidents. Our trauma proportion of 60% places our findings within the range of these studies but closer to Akinpelu et al. [10], possibly reflecting similar environmental factors, including roadside injuries and occupational exposure.

Hypertension accounted for 20% of our cases, consistent with the meta-analysis conducted by Min HJ et al., who reported that the risk of epistaxis significantly increased in patients with hypertension (OR: 1.532) [11]. However, Sharma et al. in an elderly cohort reported hypertension as a significantly higher etiological factor at 59.2%, underscoring that hypertension becomes a dominant etiology with increasing age [6].

6% of our cases were due to dengue fever, which is endemic to this geographical area. Literature review showed similar studies presenting with epistaxis [12,13]. Due to its high prevalence in this area, increased public awareness regarding the source and transmission of this disease must be made at the grassroots level for adequate prevention and control of this disease, as it is totally preventable. Although community health services are hard at work in this regard, much progress has yet to be made in mitigating and controlling the disease. Sentinel surveillance and stringent control measures have to be ensured by the relevant health authorities.

The therapeutic significance of antiplatelet medication as a modifiable risk factor was highlighted by the 18 cases (4.7%) in our study. Six of these patients were on aspirin monotherapy, eight were on aspirin and clopidogrel dual antiplatelet medication, and four had a history of exposure to combination antiplatelet regimens. Antiplatelet medications, especially clopidogrel and aspirin, work by permanently inhibiting platelet aggregation, which compromises primary hemostasis and puts patients at risk for mucosal bleeding. Antiplatelet and anticoagulant therapy has been shown in prior research to be significantly associated with an increased risk of hospital-based interventions and an increased frequency, severity, and recurrence of epistaxis [14,15]. This emphasizes the significance of a thorough medication history evaluation and interdisciplinary collaboration in striking a balance between thromboembolic protection and hemorrhagic risk during treatment.

Idiopathic cases in our study accounted for 4.7%, lower than those reported by Islam et al. and Gilyoma et al. (25% and 26.9%) [4,5], which may indicate more comprehensive diagnostic evaluations at our center.

In our cohort, recurrent bleeding was documented in 25% of patients, and associated symptoms such as headache, nasal obstruction, and generalized fatigue were also recorded (Table 3). Kumar et al. [8] reported nasal obstruction (72.6%), nasal discharge (53.42%), headache (42.47%), and pain over the nose (34.25%), while Ruhela et al. [16] reported nasal obstruction in 44.44% of cases, indicating that symptom profiles vary across populations and underlying etiologies.

The management strategies employed in our center showed that anterior nasal packing with commercially available 10 cm polyvinyl alcohol (Merocel) nasal pack was the most common modality (59.9%), followed by conservative management (26.6%), combined (anterior packing with Merocel along with posterior sterile gauze roll pack) packing (6.3%), cauterization with 15% W/W of silver nitrate (5.9%), and surgical ligation of external carotid artery by open cervical approach (1.3%). Among the 6.3% of patients who required combined packing, three required blood transfusion. This strongly corresponds with previous findings: Sharma et al. noted that anterior nasal packing with merocele was the predominant intervention (50%), Islam et al. documented anterior packing in 82.69% of patients and posterior packing in 2.89%, Gilyoma et al. found conservative management alone effective in 40.4%, followed by anterior packing in 38.5%, emphasizing that nasal packing was adequate in most cases. Thus, consistent with previous literature, nasal packing remains a cornerstone of epistaxis management, particularly in resource-limited settings [4-6].

The treatment outcomes in our study demonstrated a high initial success rate of 93.7%, with only 6.3% requiring repeat intervention. This aligns with the historically reported success rates of conservative management at similar tertiary centers [4-6]. Complication rates in our study were low, with rebleeding in 6%, bilateral maxillary and ethmoidal sinusitis in 4%, and septal ulceration in 2%. These rates compare favorably to the 3.8% complication rate observed by Gilyoma et al., suggesting that conservative measures adopted at our institution are both safe and effective.

In our cohort, trauma-related epistaxis was most commonly managed with anterior packing (59.9%) and conservative management (26.6%). Combined packing was used in 6.3% of cases, including some patients with hypertension. As no inferential analyses were performed, these treatment patterns are reported descriptively and not as comparative associations.

Idiopathic and infection-related cases were generally managed conservatively or with anterior packing, similar to patterns observed in other studies [4,5].

Overall, when comparing our findings with established literature, several patterns remain consistent: a significant male predominance, trauma as a leading cause, anterior epistaxis in the majority of cases, and nasal packing as the primary treatment modality. The similarities across studies conducted in India, Bangladesh, Tanzania, and Nigeria highlight the global relevance of these patterns despite varying demographic structures [3-9,16]. Minor variations in etiological proportions, symptom profiles, and hospital stay durations can largely be attributed to population characteristics, healthcare access, and institutional practice variations. This study adds to the growing body of evidence emphasizing the importance of early identification of etiological factors - particularly trauma and hypertension - and highlights that most cases can be effectively managed with conservative measures, achieving high success rates and minimal complications.

## Conclusions

This study highlights that epistaxis continues to be an all-time ENT emergency. The low rates of complications and need for re-intervention further affirm the effectiveness of established management protocols in a tertiary care setting. Enhancing preventive strategies, especially those focused on minimizing trauma and improving hypertension control, alongside prompt clinical management, can substantially reduce the morbidity associated with epistaxis.
